# Mitochondrial alterations as potential early events in neuromuscular junction remodelling with muscle disuse

**DOI:** 10.1113/EP093006

**Published:** 2025-07-28

**Authors:** Evgeniia Motanova, Evgenii Lysenko, Fabio Sarto, Mladen Gasparini, Boštjan Šimunic, Rado Pišot, Marco V. Narici

**Affiliations:** ^1^ Department of Biomedical Sciences University of Padua Padua Italy; ^2^ Department of Biology University of Padua Padua Italy; ^3^ Centre of Studies and Activities for Space “Giuseppe Colombo” University of Padua Padua Italy; ^4^ Izola General Hospital Izola Slovenia; ^5^ Science and Research Center Koper Institute for Kinesiology Research Koper Slovenia; ^6^ CIR‐MYO Myology Center University of Padua Padua Italy

**Keywords:** disuse, mitochondria, neuromusclular junction, skeletal muscle

## Abstract

Physical inactivity impairs neuromuscular health, promoting skeletal muscle atrophy, mitochondrial changes, and neuromuscular junction (NMJ) instability. However, the interplay between mitochondria and NMJ alterations in the context of muscle disuse remains underexplored. To investigate whether mitochondrial alterations may precede NMJ remodelling during short‐term physical inactivity, we recruited nine healthy young males who underwent 10 days of bed rest. Vastus lateralis muscle biopsies and blood samples were collected at baseline and at the end of bed rest. Mitochondrial protein content and morphology were assessed via western blotting, blue native PAGE and transmission electron microscopy. Circulating C‐terminal agrin fragment (CAF) was used as an indirect biomarker for NMJ remodelling. Despite no significant changes in mitochondrial oxidative phosphorylation complexes and supercomplexes, mitochondrial morphology and volume density in skeletal muscle fibres, we observed an increase in phosphorylated DRP1 S637 and trends toward reduced mitofusins 1 and 2, indicating a potential early shift in fission–fusion dynamics. Circulating CAF concentration remained unchanged. Interestingly, although limited by small sample size, rare electron microscopy images from one participant revealed features suggestive of altered mitochondrial phenotype in motor axon terminals at the NMJ after bed rest. While clearly preliminary and qualitative, these observations raise the possibility of early mitochondrial changes in the presynaptic terminal during disuse. We emphasise the speculative nature of this finding and stress the need for further research using larger, targeted datasets to explore this hypothesis more rigorously.

## Abstract (highlights)

- **What is the central question of this study?**
  Is short‐term bed rest associated with early mitochondrial changes in motor axon terminals at the neuromuscular junction (NMJ), potentially preceding overt NMJ remodelling?
- **What is the main finding and its importance?**
  Qualitative electron microscopy images from a single participant revealed altered mitochondrial morphology in NMJ motor neuron terminals after 10 days of bed rest. While preliminary and limited in scope, these findings raise the hypothesis that presynaptic mitochondrial phenotype may change before detectable NMJ instability.

## 1 INTRODUCTION

Physical inactivity, such as bed rest, leads to a significant deterioration of the neuromuscular system. This includes muscle atrophy (Marusic et al., 2021), impaired mitochondrial function (Eggelbusch et al., 2024; Noone et al., 2023; Standley et al., 2020), and instability of the neuromuscular junction (NMJ) (Monti et al., 2021; Motanova et al., 2025; Sarto et al., 2025). The NMJ, a specialised excitatory synapse between motor neurons and skeletal muscle fibres, is essential for facilitating muscle contraction and maintaining muscle function. Mitochondria play a pivotal role in both skeletal muscle fibres and motor neuron terminals. In muscle fibres, they generate ATP necessary for contraction and metabolic homeostasis. In motor neuron terminals, mitochondria supply the energy required for neurotransmitter release and other processes vital for effective synaptic transmission at the NMJ (Motanova et al., 2024).

While the impact of inactivity on skeletal muscle mitochondria is well‐documented, the specific effects on mitochondria within motor neuron terminals at the NMJ and their interplay during disuse remain poorly understood. Given the NMJ's reliance on mitochondrial function for synaptic transmission (Harris et al., 2012), it is plausible that mitochondrial alterations in motor neurons could play a role and contribute to NMJ remodelling during periods of reduced physical activity.

In this study, we investigated early mitochondrial and NMJ alterations following 10 days of bed rest in healthy young adult males. By analysing skeletal muscle mitochondrial protein expression and morphology, circulating C‐terminal agrin fragment (CAF) as an indirect marker of NMJ integrity, and rare electron microscopy images of human NMJs, we aimed to explore the hypothesis that mitochondrial alterations at the motor neuron terminal may contribute to early stages of NMJ remodelling during physical inactivity.

## 2 METHODS

### 2.1 Ethical approval

The study followed the *Declaration of Helsinki* and was approved by the Slovenian Ministry of Health (21 July 2023; ref. 0120‐123/2023/9). All participants provided written informed consent and could withdraw at any time.

### 2.2 Participants

Nine healthy young males (age: 22.8 ± 4.4 years; height: 1.83 ± 0.06 m; body mass: 79.1 ± 6.7 kg) volunteered for this study. Exclusion criteria included smoking, drug use, clotting disorders, previous deep vein thrombosis, cardiovascular, metabolic, musculoskeletal, neurological and psychiatric conditions, professional sports involvement, and ferromagnetic implants.

### 2.3 Experimental protocol

This study is part of a larger 21‐day horizontal bed rest campaign (Sarto et al., 2025) designed with three data collection time points: at baseline (before bed rest, BR0), after 10 days (BR10) and after 21 days of bed rest. The present study specifically focuses on the data collected at BR10. Muscle biopsies and blood samples were collected at BR0 and BR10. During the intervention, participants remained on strict horizontal bed rest under continuous medical supervision, with no upright posture or exercise allowed.

### 2.4 Skeletal muscle tissue collection

Skeletal muscle samples of the vastus lateralis (∼150 mg) were collected via biopsy using a Weil–Blakesley conchotome (Gebrüder Zepf Medizintechnik GmbH, Dürbheim, Germany) under local anaesthesia (2% lidocaine) at BR0 and BR10, as previously described (Motanova et al., 2025; Sarto et al., 2025). To avoid repeated sampling from the same site, the BR0 biopsy was taken from the right leg, and the BR10 biopsy was taken from the left leg. Biopsies were performed in the morning (09.00 h), in a fasted state, for both time points.

Immediately after collection, samples were dissected free of fat and connective tissue and divided into different portions:

- Two ∼20 mg portions were snap‐frozen and stored at −80°C for western blot and blue native PAGE analyses.
- A 5 mg portion was fixed overnight in 2.5% glutaraldehyde + 2% paraformaldehyde in 0.1 M sodium cacodylate buffer, then stored in a 0.1 M sodium cacodylate buffer at 4°C until processing for transmission electron microscopy (TEM).

### 2.5 Blood collection

Venous blood samples were collected from the median cubital vein at BR0 and BR10 (07.00 h) from participants in a fasted state. After allowing the samples to rest at room temperature for 10 min, they were centrifuged at 2500 *g* (corresponding to 3880 rpm) using a Centric 400 centrifuge (Domel, Železniki, Slovenia). Plasma aliquots (100 µL) were then stored in liquid nitrogen at −80°C until further analysis.

### 2.6 C‐terminal agrin fragment detection

Plasma CAF levels were quantified using ELISA kits (Human Agrin SimpleStep ELISA, Abcam, Cambridge, UK, cat. no. ab216945), following manufacturer instructions. Samples were diluted 1:6, analysed in duplicate, and read at 450 nm using a microplate reader (Tecan, Infinite M200, Männedorf, Switzerland). CAF concentrations were interpolated from standard curves and corrected for dilution. Measurement coefficients of variation were <2%.

### 2.7 Skeletal muscle lysate preparation and western blotting

Frozen muscle tissue was lysed in Pierce RIPA buffer (cat. no. 89901, Thermo Fisher Scientific, Waltham, MA, USA) containing Halt protease/phosphatase inhibitors (cat. no. 1861281, Thermo Fisher Scientific, Waltham, MA, USA). Homogenisation was performed using a Velp Scientifica LS Overhead Stirrer (Velp Scientifica, Usmate, Italy) at speed 5 (30 s ×3), followed by incubation on ice for 10 min. Lysates were centrifuged (15,200 *g*, 10 min, 4°C) in a Frontier 5000 centrifuge (FC5515R, Ohaus, Parsippany, NJ, USA). Protein concentration was determined using the Pierce BCA Assay Kit (cat. no. 23225, Thermo Fisher Scientific, Waltham, MA, USA) and a Tecan Infinite 200 Pro plate reader. Proteins were separated on either 3–8% Tris‐acetate gels (cat. no. EA03785BOX, Thermo Fisher Scientific, Waltham, MA, USA) or 4–12% Bis–Tris gels (cat. no. NP0336BOX, Thermo Fisher Scientific, Waltham, MA, USA), with the PageRuler Plus prestained ladder (cat. no. 26619, Thermo Fisher Scientific, Waltham, MA, USA) as molecular marker. Electrophoresis was performed at 4°C in an XCell SureLock Mini‐Cell (cat. no. EI0001, Thermo Fisher Scientific, Waltham, MA, USA) at 60 V for 30 min, then 100 V until completion. Proteins were transferred onto nitrocellulose membranes at 100 V for 90 min at 4°C using a Mini Trans‐Blot Cell (cat. no. 1703930, Bio‐Rad Laboratories, Hercules, CA, USA). Membranes were blocked with 5% milk or 5% BSA (1 h, room temperature), followed by incubation with primary antibodies: mouse anti‐total oxidative phosphorylation (OXPHOS) cocktail (NDUF88, COX II, SDHB, UQCRC2, ATP5A; 1:1000, cat. no. Ab110411, Abcam), rabbit anti‐OPA1 (1:700, cat. no. Ab42364, Abcam, Cambridge, UK), mouse anti‐DRP1 (1:1000, 611113, BD Biosciences, San Jose, CA, USA), rabbit anti‐phospho‐DRP1 (Ser637; 1:500, cat. no. 4867, Cell Signaling Technology, Danvers, MA, USA), mouse anti‐MFN2/MFN1 cocktail (1:1000, cat. no. Ab57602, Abcam, Cambridge, UK), and rabbit anti‐β‐actin (1:1000, ab8227, Abcam, Cambridge, UK). For OXPHOS protein detection, samples were heated to 37°C prior to loading, as recommended by the manufacturer. For all other targets, samples were boiled at 95°C for 5 min before electrophoresis. After primary antibody incubation, membranes were treated with horseradish peroxidase (HRP)‐conjugated secondary antibodies: anti‐mouse (1:2000, cat. no. GTX213111‐01, GeneTex, San Antonio, TX) or anti‐rabbit (1:2000, cat. no. GTX213110‐01, GeneTex, San Antonio, TX) for 1 h at room temperature. Detection was performed using Immobilon Crescendo HRP substrate (cat. no. WBLUR0500, Millipore, Billerica, MA, USA) and visualized with an iBright FL1500 system (cat. no. A44241, Thermo Fisher Scientific, Waltham, MA, USA). Densitometry was analysed using ImageJ (NIH, Bethesda, MD, USA), with protein bands normalised to β‐actin as loading control.

### 2.8 Mitochondrial isolation and blue native PAGE

Mitochondria were isolated from ∼20 mg skeletal muscle using digitonin permeabilisation and differential centrifugation (Fernandez‐Vizarra & Zeviani, 2021; Frezza et al., 2007). Homogenisation was done with the Velp LS Overhead Stirrer at speed 5 (15 s, ×2) in isolation buffer 1 (67 mM sucrose, 50 mM Tris–HCl, 50 mM KCl, 10 mM EDTA, and 0.2% BSA; pH 7.4). After sequential centrifugations (700 *g* then 10,000 *g*, 10 min, 4°C; Ohaus Frontier 5000 FC5515R, Parsippany, NJ, USA), the pellet was resuspended in isolation buffer 2 (250 mM sucrose, 3 mM EGTA/Tris, and 10 mM Tris–HCl; pH 7.4). Protein concentration was measured with the Pierce Bradford Plus Assay (cat. no. 23238, Thermo Fisher Scientific). For blue native (BN)‐PAGE, 30 µg mitochondrial protein was solubilised with digitonin (4 mg/mg protein), mixed with NativePAGE Sample Buffer (cat. no. BN2003, Thermo Fisher Scientific, Waltham, MA, USA), centrifuged (15,200 *g*, 30 min), and loaded on NativePAGE gels (cat. no. BN1001BOX, Thermo Fisher Scientific, Waltham, MA, USA). Electrophoresis was performed at 100 V (1 h), then 250 V (2.5 h, 4°C) with NativePAGE Running Buffer Kit (cat. no. BN2007, Thermo Fisher Scientific, Waltham, MA, USA). NativeMark Protein Standard (cat. no. 57030, Thermo Fisher Scientific, Waltham, MA, USA) served as marker. Western blotting followed the protocol above. Supercomplexes were probed using antibodies against complex I subunit NDUFS1 (1:1000, cat. no. Ab169540, Abcam, Cambridge, UK) and complex II subunit SDHA (1:1000, cat. no. Ab14715, Abcam, Cambridge, UK), with HRP‐conjugated secondary antibodies from GeneTex (anti‐rabbit cat. no. GTX213110‐01; anti‐mouse cat. no. GTX213111‐01, GeneTex, San Antonio, TX). Quantification of supercomplexes was based on NDUFS1 immunodetection that was normalised to complex II SDHA subunit to correct for loading differences (Vogel et al., 2007).

### 2.9 Transmission electron microscopy

Sample processing was performed at the Imaging Facility, Department of Biology, University of Padova (Italy). Muscle samples were fixed in glutaraldehyde and paraformaldehyde, post‐fixed with osmium tetroxide, dehydrated in ethanol series, and embedded in epoxy resin (EMbed 812, Delta Microscopies, Mauressac, France). Ultrathin sections were obtained (Leica EM UC7 ultramicrotome, Wetzlar, Germany), stained with uranyl acetate and lead citrate, and imaged using a Tecnai G2 TEM (FEI Company, Hillsboro, OR, USA) at 120 kV. Digital images were captured at ×13,000 magnification, targeting subsarcolemmal (SS) and intermyofibrillar (IMF) mitochondria. Two blinded operators analysed mitochondrial volume density and morphology. Mitochondrial volume density was quantified by point counting using a 150 nm grid (2500 vertices grid), expressed per myofibrillar space (IMF, µm^3^/µm^3^) or surface area (SS, µm^3^/µm^2^). Total mitochondrial volume density was calculated as IMF + (SS/20). Morphology was assessed by tracing mitochondria in ImageJ v1.52v, measuring aspect ratio and form factor to evaluate length‐to‐width ratio and complexity (Gamboa & Andrade, 2010; Jacobs et al., 2016; Picard et al., 2013).

Additionally, images of NMJs were acquired for illustrative purposes following the same TEM sample preparation and imaging protocols. However, due to the identification of only four NMJs (two at BR0 and two at BR10), all from a single participant, no quantitative analysis was performed.

### 2.10 Statistical analysis

Data normality was assessed using the Shapiro–Wilk test and Q–Q plots. Non‐normally distributed variables were analysed using the Wilcoxon test. Significance was set at *P* ≤ 0.05. Effect sizes were calculated using Hedges’ *g*, an adjusted version of Cohen's *d* that corrects for small sample bias by applying a correction factor to the standardised mean difference. Effect sizes were interpreted as small (≤0.2), medium (0.2–0.5), or large (≥0.8). Analyses were performed using GraphPad Prism (version 8.0.1 for Windows, GraphPad Software, San Diego, CA, USA) and Microsoft Excel (Version 2412 Build 16.0.18324.20092). Graphs were generated with GraphPad Prism.

## 3 RESULTS

All participants completed the bed rest intervention, and there were no documented side effects associated with either the bedrest or the biopsy and blood collection procedures.

### 3.1 Ten‐day bed rest does not induce alteration of mitochondrial oxidative phosphorylation complexes and supercomplexes

**FIGURE 1:**
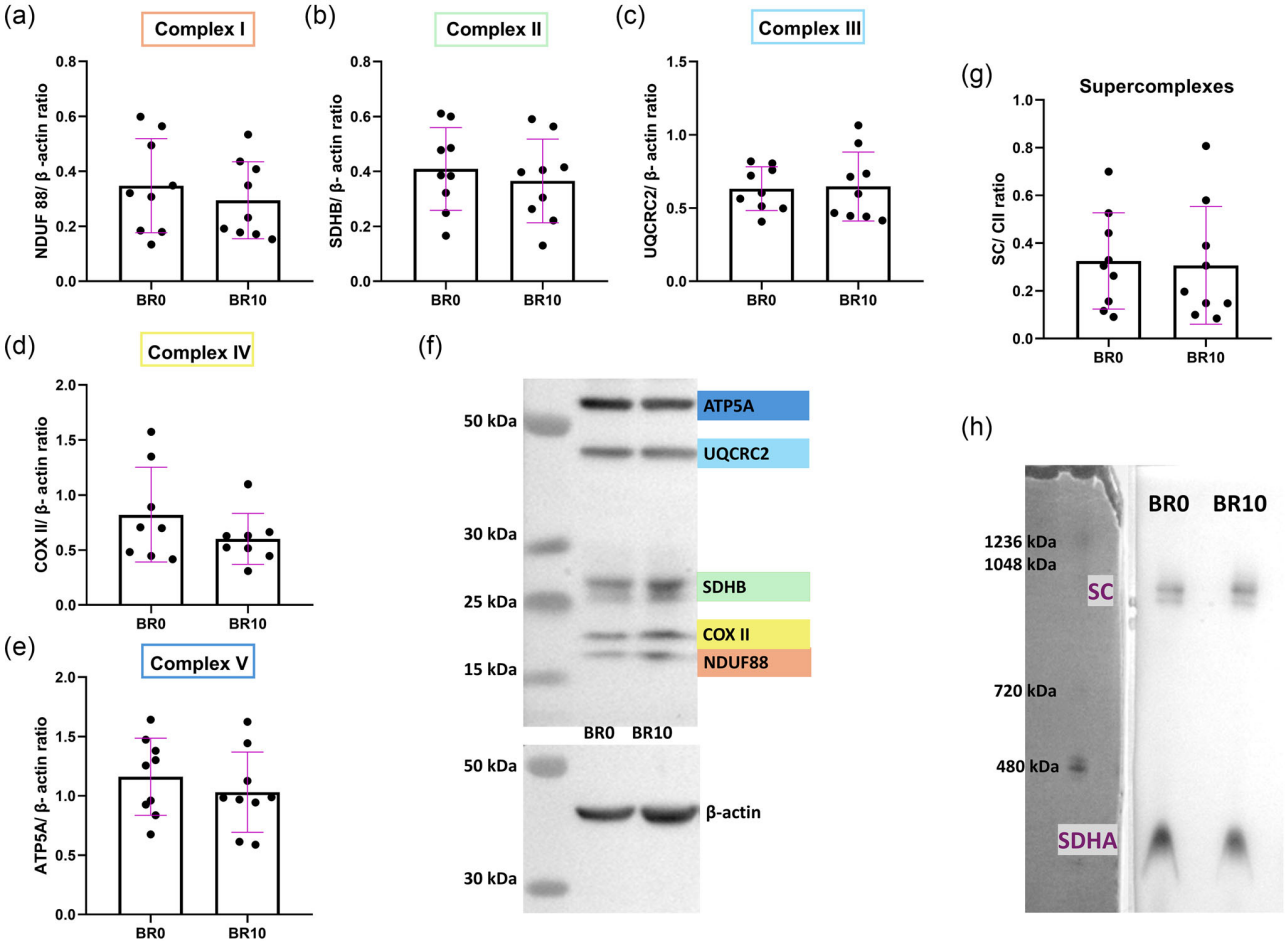
Abundance of mitochondrial oxidative phosphorylation (OXPHOS) complexes and supercomplexes. (a–e) Protein abundance of individual mitochondrial electron transport chain components: NDUFB8 (complex I), SDHB (complex II), UQCRC2 (complex III), COX II (complex IV), and ATP5A (complex V), all normalised to β‐actin. (f) Representative western blot of OXPHOS proteins and β‐actin from one participant showing protein expression at baseline (BR0) and after 10 days of bed rest (BR10). (g) Quantification of mitochondrial supercomplex abundance normalised to complex II (SDHA). (h) Representative blue native PAGE blot showing mitochondrial supercomplexes and complex II (SDHA) from one participant showing protein expression at BR0 and BR10. *n *= 9, except for (d), where *n *= 8.

To explore potential changes in mitochondrial protein abundance after bed rest, we analysed the levels of mitochondrial oxidative phosphorylation (OXPHOS) complexes and supercomplexes (Figure 1a–h). Specifically, we assessed the levels of proteins within each complex: NADH dehydrogenase (ubiquinone) 1 β subcomplex subunit 8 (NDUFB8) for complex I, succinate dehydrogenase subunit B (SDHB) for complex II, ubiquinol–cytochrome *c* reductase (UQCRC2) for complex III, cytochrome *c* oxidase subunit II (COX II) for complex IV, and α‐subunit of mitochondrial ATP synthase (ATP5A) for complex V. No changes were observed in the abundance of these proteins at BR10: NDUFB8 (*P* = 0.7344; Hedges’ *g* = 0.30), SDHB (*P* = 0.3008; Hedges’ *g* = 0.24), UQCRC2 (*P* = 0.0625; Hedges’ *g* = 0.07), COX II (*P* = 0.1094; Hedges’ *g* = 0.38), and ATP5A (*P* = 0.0977; Hedges’ *g* = 0.36). (Figure 1a–f)

Similarly, the analysis of mitochondrial supercomplexes containing complex I revealed no significant alterations at BR10 (*P* = 0.3008; Hedges’ *g* = 0.036) (Figure 1g, h). Complex I protein was selected for detection because it is a component of all major mitochondrial supercomplexes (Jha et al., 2016).

### 3.2 Fission and fusion protein changes without mitochondrial morphology alterations

**FIGURE 2:**
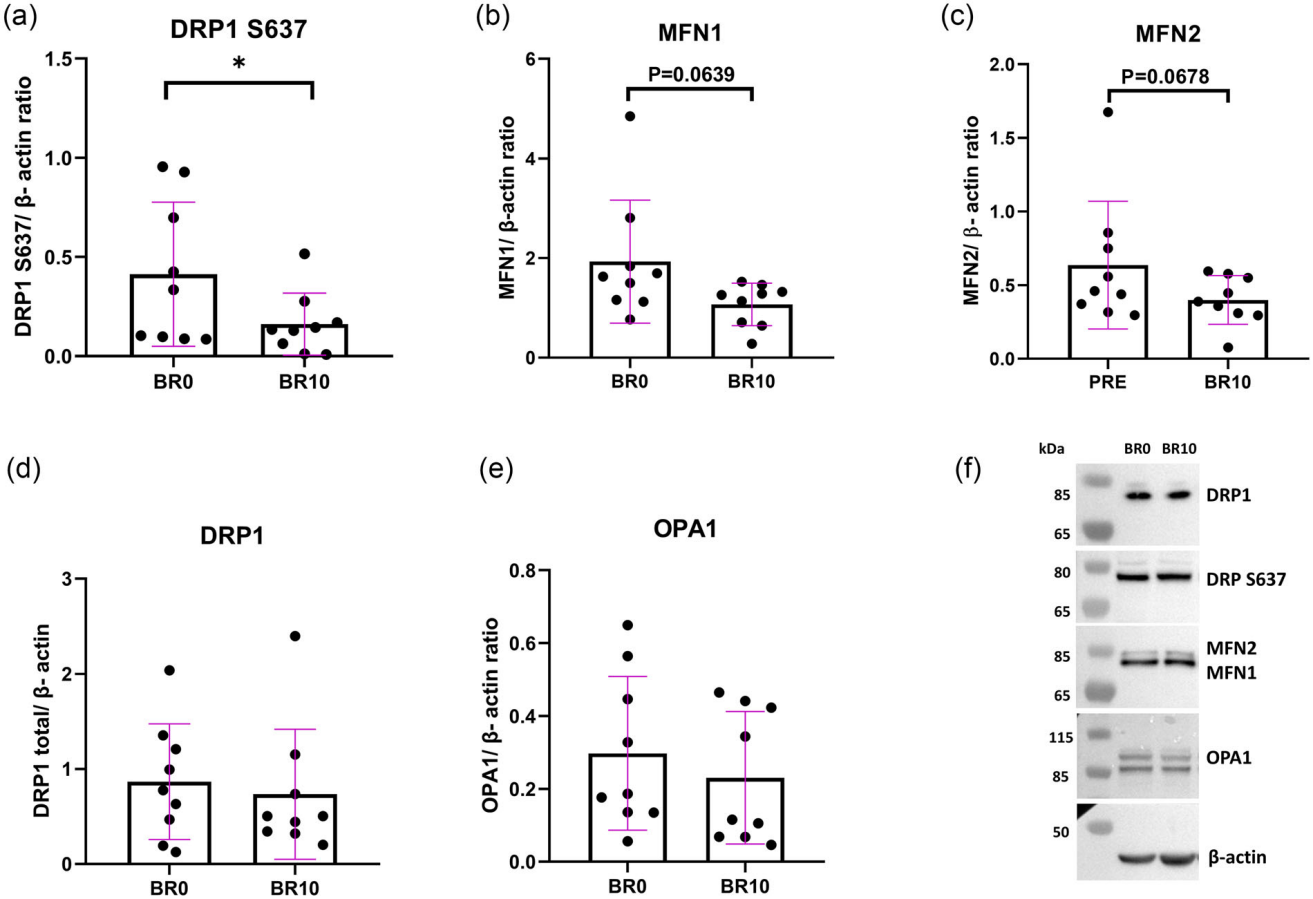
Abundance of mitochondrial fission and fusion‐related proteins. (a) Protein levels of DRP1 phosphorylated at Ser637, (b) mitofusin 1, (c) mitofusin 2, (d) total DRP1, and (e) OPA1, all normalized to β‐actin. (f) Representative western blots from one participant showing protein expression of these proteins at baseline (BR0) and after 10 days of bed rest (BR10). *n* = 9.

We then examined expression levels of proteins involved in the mitochondrial fission and fusion machinery (Figure 2a–f). We observed a decrease in phosphorylated dynamin‐related protein 1 (DRP1) at serine 637 (*P* = 0.0391; Hedges’ *g* = 0.78) (Figure 2a). Additionally, there was a trend towards reduced levels of mitofusin 1 (*P* = 0.0639; Hedges’ *g* = 0.83) and mitofusin 2 (*P* = 0.0678; Hedges’ *g* = 0.61) (Figure 2b, c), although no changes were detected for optic atrophy 1 (OPA1) (*P* = 0.4258; Hedges’ *g* = 0.29) (Figure 2d) or total DRP1 (*P* = 0.5703; Hedges’ *g* = 0.17) (Figure 2e)

**FIGURE 3:**
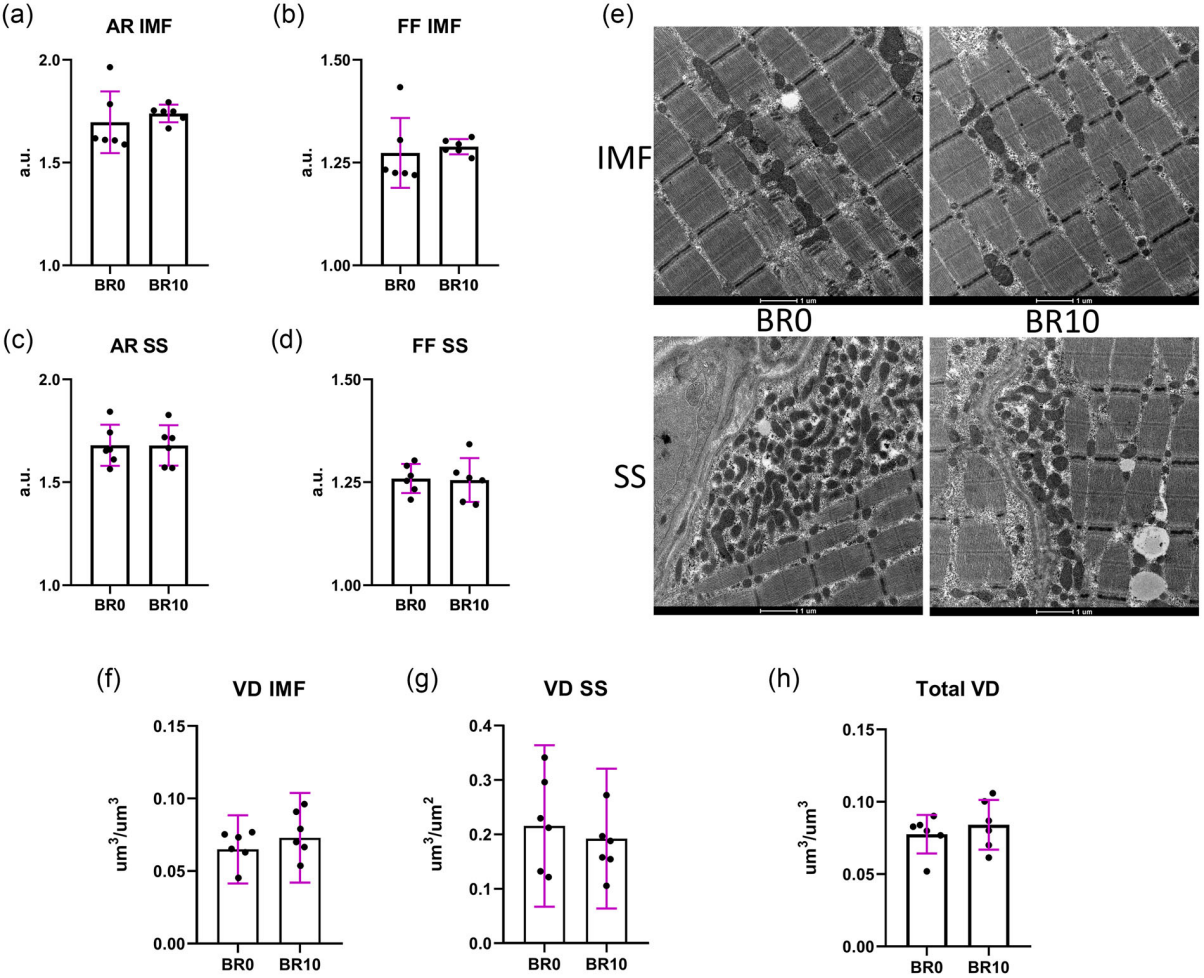
Mitochondrial morphology and volume density based on transmission electron microscopy. (a) Aspect ratio (AR) of intermyofibrillar (IMF) mitochondria. (b) Form factor (FF) of IMF mitochondria. (c) AR of subsarcolemmal (SS) mitochondria. (d) FF of SS mitochondria. (e) Representative TEM images of IMF (top) and SS (bottom) mitochondria before (BR0) and after bed rest (BR10); scale bar = 1 µm. (f) Volume density (VD) of IMF mitochondria. (g) VD of SS mitochondria. (h) Total mitochondrial VD across both compartments. *n *= 6.

To investigate whether these protein changes translated into alterations in mitochondrial morphology in the skeletal muscle fibres (Figure 3a–h), we first analysed aspect ratio and form factor – key descriptors of mitochondrial shape of IMF and SS mitochondrial fractions. However, no differences were found in these morphological parameters after bed rest: aspect ratio IMF (*P* = 0.5625; Hedges’ *g* = 0.24), form factor IMF (*P* = 0.4375; Hedges’ *g* = 0.13), aspect ratio SS (*P *> 0.9999; Hedges’ *g* = 0.006), form factor SS (*P* = 0.6875; Hedges’ *g* = 0.07).

We further assessed mitochondrial volume density, which remained unchanged at BR10 for IMF (*P* = 0.5073; Hedges’ *g* = 0.25) (Figure 3f), SS (*P* = 0.5625; Hedges’ *g* = 0.16) (Figure 3g) and total (*P* = 0.6875; Hedges’ *g* = 0.13) (Figure 3h).

### 3.3 Qualitative observations of mitochondria at the presynaptic NMJ terminal

**FIGURE 4:**
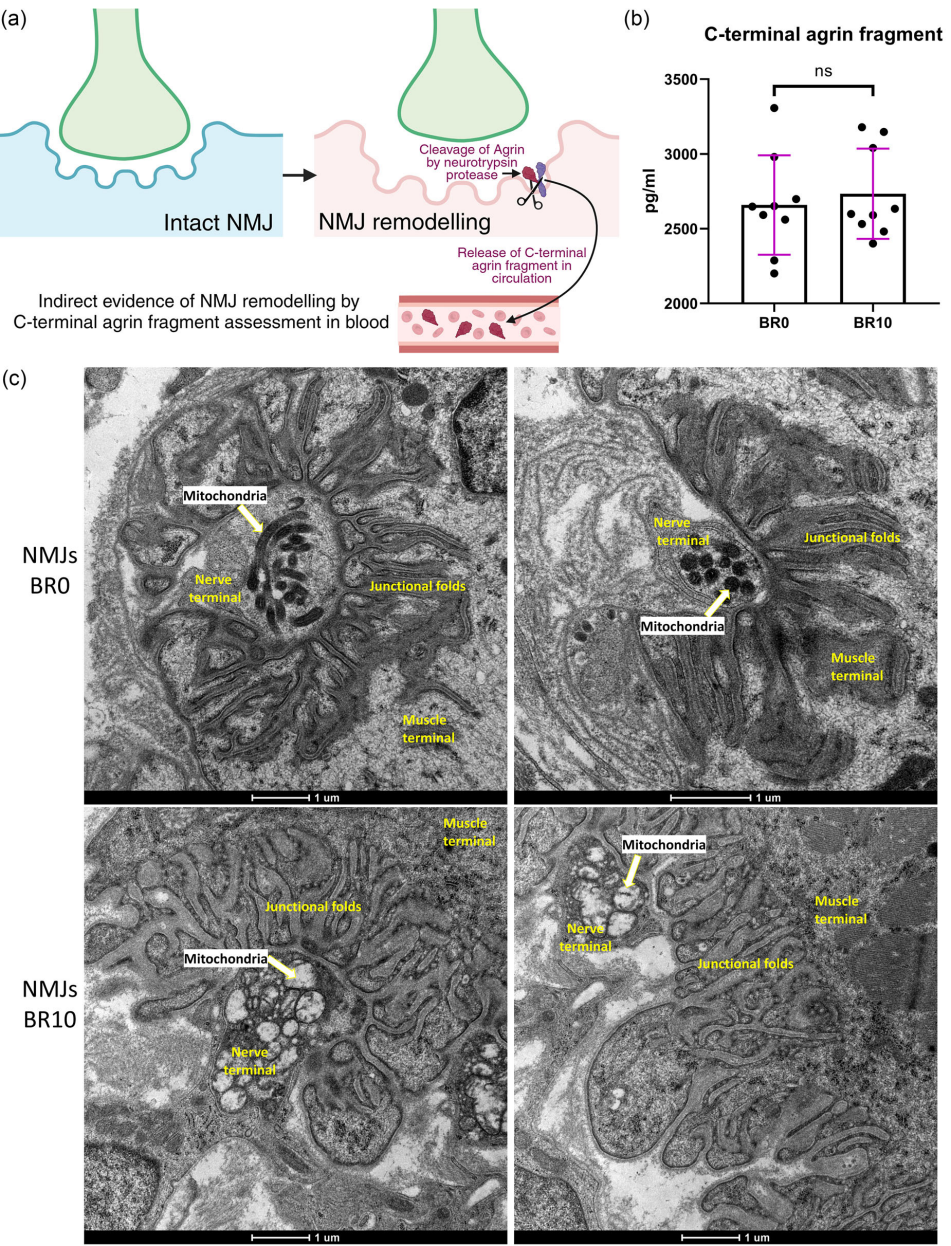
Circulating C‐terminal agrin fragment (CAF) and neuromuscular junction structure. (a) Schematic representation of the cleavage of agrin resulting in the release of the C‐terminal agrin fragment (CAF) during neuromuscular junction (NMJ) remodelling. (b) Circulating CAF protein levels measured before (BR0) and after 10 days of bed rest (BR10). *n* = 8 (c) Representative transmission electron microscopy images of NMJs at BR0 and BR10, highlighting structural changes of mitochondria within the motor neuron NMJ terminals; scale bar = 1 µm. *n* = 1.

Considering the observed mitochondrial changes in skeletal muscle, we explored whether early signs of bed rest‐induced disuse might be reflected at the NMJ. First, as an indirect proxy for NMJ remodelling, we measured circulating C‐terminal agrin fragment concentrations (Figure 4a) in plasma. However, no significant differences were found after bed rest (*P* = 0.2500; Hedges’ *g* = 0.17) (Figure 4b).

In addition, we obtained electron microscopy images of NMJs from tissue samples collected at BR0 and BR10. A major limitation in studying human NMJs is the difficulty in obtaining NMJ‐positive tissue from muscle biopsies (Motanova et al., 2024, 2025; Sarto et al., 2025). In this study, we were able to identify two NMJs at BR0 and two at BR10, all from the same participant. Due to this limited yield, we did not perform a quantitative analysis.

Despite this constraint, qualitative inspection of the available images suggested potential alterations in mitochondrial morphology at the motor neuron terminal following bed rest. At BR0, mitochondria appeared electron‐dense and structurally intact. At BR10, they appeared swollen and rounded, with disrupted or less defined cristae – features indicative of structural impairment (Figure 4c). While these observations are intriguing and may indicate early mitochondrial alterations at the presynaptic NMJ terminal, we emphasize that the data are preliminary and derived from a single participant. A substantially larger sample size will be required to validate and generalize these findings.

## 4 DISCUSSION

In this study, we investigated early mitochondrial alterations after 10 days of bed rest, with the aim of exploring potential early contributors to disuse‐induced NMJ remodelling. Our results indicate that mitochondrial protein abundance and morphology remain largely unchanged after 10 days of disuse in skeletal muscle. Specifically, we observed no differences in the expression of OXPHOS complexes or mitochondrial supercomplexes, which may suggest the absence of overt mitochondrial dysfunction, in line with previous evidence suggesting that 10 days of bed rest do not alter mitochondrial respiration in permeabilised vastus lateralis muscle fibres (Zuccarelli et al., 2021).

However, the presence of reduced levels of phosphorylated DRP1 S637 – a modification shown to inhibit DRP1 GTPase activity and modulate its association with the mitochondrial membrane (Sun et al., 2025) – suggests a potential increase in fission activity. Nevertheless, this alone is not determinative, as mitochondrial fission is regulated by multiple interacting factors (Yu et al., 2019). Additionally, we observed trends toward reduced expression of mitofusin 1 and 2, further supporting a potential shift toward fission over fusion. Despite these molecular indicators, such changes were not sufficient to alter mitochondrial morphology or volume density, indicating that not just the content of skeletal muscle mitochondria but also the mitochondrial structure remains largely preserved.

On the NMJ side, the circulating concentration of CAF – a commonly used biomarker of NMJ remodelling (Monti et al., 2023) – was not changed suggesting that the NMJ remained intact following 10 days of bed rest. No systemic signs of NMJ instability were evident after 10 days of bed rest. While CAF offers a practical window into NMJ remodelling, it remains an indirect proxy and does not provide mechanistic or structural detail on NMJ integrity (Monti et al., 2023; Pratt et al., 2021). In this context, the lack of quantitative NMJ morphological or functional assessments in this study limits the conclusions that can be drawn regarding the degree or presence of NMJ remodelling.

To the authors’ knowledge, no short‐term bed rest studies have assessed in vivo NMJ function electrophysiologically in young individuals. However, a 10‐day unilateral limb suspension period did not induce any functional alterations of the NMJ (i.e., no changes in its trasmission properties) (Sarto et al., 2022), further supporting the idea that short‐term disuse may not be sufficient to induce overt NMJ functional decline in the young individuals.

Despite the minimal changes observed in skeletal muscle mitochondria, electron microscopy images from one participant revealed altered mitochondrial ultrastructure within motor neuron terminals at the NMJ. Due to the limited availability of NMJ‐positive tissue from muscle biopsies, these observations are qualitative and not generalisable. Nonetheless, mitochondria at BR10 appeared swollen with disrupted cristae, contrasting with the dense, structured mitochondria observed at BR0. These images may offer early insight into the potential vulnerability or altered phenotype of presynaptic mitochondria following disuse.

We emphasise that these NMJ‐related findings are preliminary, qualitative and derived from a single participant. However, they raise the hypothesis that presynaptic mitochondrial modulation or stress may occur early, potentially preceding structural or functional NMJ remodelling. Given the rarity of such data in humans – and the well‐known challenge of capturing NMJs in biopsy‐derived tissue (Motanova et al., 2024), we believe these findings provide a valuable starting point for future investigations. Further work – both in preclinical models and well‐powered human studies – is needed to substantiate and expand upon this possibility.

This hypothesis is reinforced by findings we previously reported from the same study at a later timepoint (21 days of bed rest). At this stage, we observed an increase in NMJ denervation, assessed morphologically by evaluating the overlap between pre‐ and postsynaptic terminals and categorising NMJs based on this overlap. Alongside this, we found elevated circulating CAF levels and impaired NMJ transmission properties, further supporting the potential of CAF as an indirect marker of NMJ remodelling (Sarto et al., 2025).

Taken together, these findings raise the possibility that mitochondrial changes within motor neuron terminals could represent one of the early events in a sequence contributing to NMJ remodelling during disuse. This idea aligns with previous evidence from animal models of ageing, in which presynaptic mitochondrial alterations were implicated in NMJ instability (García et al., 2013). Given that skeletal muscle mitochondrial changes were minimal and CAF levels were unaltered at BR10, the observed mitochondrial changes at the presynaptic terminal raise the possibility that neuronal mitochondrial dysfunction may contribute to the initiation of NMJ instability. We stress that this interpretation is highly preliminary, based on a small number of NMJ‐positive samples, and should be viewed as hypothesis‐generating rather than conclusive.

### 4.1 Limitations and future directions

This study has five main limitations. First, only male participants were included, due to the higher absolute risk of first deep venous thrombosis in young women – a risk that is further increased by prolonged inactivity (Roach et al., 2014). Given the 21‐day total duration of the bed rest intervention and the fact that the current study presents only the intermediate (10‐day) time point of this longer protocol, the exclusion of female participants was based on ethical considerations. Second, the overall sample size was small, which limits the generalisability of our findings. Third, our observation regarding mitochondrial alterations within the motor neuron terminal is based on a limited number of NMJ electron microscopy images. While these qualitative observations are compelling, they highlight the need for future animal and human studies specifically designed to investigate mitochondrial involvement in the neural component of the NMJ – either through models that allow isolation of neural and muscular compartments or through larger participant cohorts that enable the acquisition of more extensive imaging datasets. Third, we did not assess NMJ morphology in this study. Thus, the ‘time course comparison’ is based solely on changes in CAF, which is an indirect way to assess NMJ remodelling. Using indirect markers like circulating CAF to assess NMJ status is common in human studies due to the difficulty of obtaining muscle biopsies with NMJs. Collecting such samples is technically challenging and limited by ethical and logistical constraints. While indirect measures offer useful information, direct morphological or functional assessments of NMJs are needed in future research to draw stronger conclusions about neuromuscular health (Jones et al., 2024). Lastly, as our study was conducted exclusively in young healthy males, it remains to be determined whether these findings generalise to females, older adults or clinical populations. Future studies including these groups are needed to explore potential differences in neuromuscular responses to disuse.

## AUTHOR CONTRIBUTIONS

Conceptualisation: Marco V. Narici, Rado Pišot, Boštjan Šimunic. Methodology: Evgeniia Motanova, Evgenii Lysenko, Fabio Sarto. Data analysis: Evgeniia Motanova, Evgenii Lysenko, Fabio Sarto. Writing – original draft: Evgeniia Motanova. Writing – review and editing: Evgeniia Motanova, Evgenii Lysenko, Fabio Sarto, Marco V. Narici, Rado Pišot, Boštjan Šimunic Project administration, Marco V. Narici, Boštjan Šimunic, Rado Pišot. Funding acquisition: Marco V. Narici, Evgeniia Motanova. Resources: Marco V. Narici, Boštjan Šimunic, Rado Pišot. Supervision: Marco V. Narici. All authors reviewed and revised the final version of the manuscript. All authors have read and approved the final version of this manuscript and agree to be accountable for all aspects of the work in ensuring that questions related to the accuracy or integrity of any part of the work are appropriately investigated and resolved. All persons designated as authors qualify for authorship, and all those who qualify for authorship are listed.

## CONFLICT OF INTEREST

None declared.

## Data Availability

Data that support the findings of this study will be made available from the corresponding author upon reasonable request.
